# Supplementary material for: Readiness of the primary health care units and associated factors for the management of hypertension and type II diabetes mellitus in Sidama, Ethiopia
Source: PeerJ. 2022 Aug 25;10:e13797. doi: 10.7717/peerj.13797 (PMC9420406; doi:10.7717/peerj.13797)
Supplement: Supplemental Information 2 [file peerj-10-13797-s002.docx]

# Annex I consent form

Questionnaire I: Cross-sectional study on DM and HTN early detection.

Hawassa University, College of Agriculture Department of Nutrition

A questionnaire on a study entitled: Service Availability and Readiness Assessment for DM and HPN, Sidama Regional State Ethiopia.

Hello Dear Sir/Madam!

My name is ------------------------, I am representing the study team coordinated and led by Tigist Kebede Mulugeta a doctoral student at Hawassa University. I am collecting data on the study entitled: Service Availability and Readiness Assessment for DM and HPN, Sidama Regional State Ethiopia.

This study will make an important contribution to Determine the readiness of the primary health care units (PHCU) to manage type II diabetes and hypertension in Sidama Regional State.

The questionnaire has three components; **I) the availability of basic amenities, the availability of trained health care providers on DM and HPN, the availability of functioning equipment, the presence of diagnostic materials and medicines (not expired or stock-out).** Your name will not be identified in relation to your personal information. For unclear questions, you are kindly requested to ask the interviewer for clarity. If you don’t feel comfortable during the interview, you are free to discontinue your participation. The data collection process will take 25 to 40 minutes. There is no direct benefit to you for participating; however, the information obtained from the study may benefit the people of our country through using it for the design of programs.

Did you understand the aim of the study? Yes ( ) No ( ).

Are you willing to represent the PHCU and participate in the study? Yes ( ) No ( ).

Signature of the participant……………………….Information
